# Factors associated with caregivers’ perceptions of advance care planning for people living with dementia

**DOI:** 10.1002/alz.087122

**Published:** 2025-01-09

**Authors:** Yuchen Zhang, Susan M Sereika, Jennifer H Lingler, Jennifer Seaman, Corinne Pettigrew, Marilyn S. Albert

**Affiliations:** ^1^ University of Pittsburgh, Pittsburgh, PA USA; ^2^ University of Pittsburgh Alzheimer’s Disease Research Center (ADRC), Pittsburgh, PA USA; ^3^ Johns Hopkins University School of Medicine, Baltimore, MD USA; ^4^ ivision of Cognitive Neuroscience, John’s Hopkins University School of Medicine, Baltimore, MD USA; ^5^ Johns Hopkins University, Baltimore, MD USA

## Abstract

**Background:**

People living with dementia (PwD) experience progressive functional decline with increasing dependence on their caregivers. Advanced care planning (ACP) has the potential to promote quality of life, reduce iatrogenic harm, and minimize overutilization of healthcare resources, yet planning ahead in the context of dementia is challenging and requires consideration of numerous factors over an extended period of time. We examined caregivers’ perceptions of current and end‐stage medical care preferences in PwD and the impact of ACP‐related discussions between caregivers and PwD.

**Method:**

This secondary analysis utilized data from the cross‐sectional survey, “Care Planning for Individuals with Dementia”, which was administered by staff at Alzheimer’s Disease Centers across the U.S. Statistical analysis employed binary, ordinal, and multinomial logistic regression. Predicting variables included caregivers’ knowledge about dementia; outcome variables were caregivers’ perceptions of PwD’s goals of medical care; covariates were relationships between caregivers and PwD, the Clinical Dementia Rating of PwD, and demographic characteristics.

**Result:**

Caregivers were mainly white (88.5%) and highly educated (mean = 15.9 years), with a mean age of 78.3 years and high dementia knowledge scores (mean = 8.4/10). Caregivers’ knowledge about dementia was associated with their perceptions about PwD’s medical care. Caregivers with higher total dementia knowledge scores had 1.32 times the odds of endorsing comfort care at end‐stage dementia (p = 0.009). Greater knowledge about severe dementia was inversely associated with perceiving the need for further discussions (knowing a lot: OR = 0.17, p<0.001; knowing some things: OR = 0.37, p = 0.006). Caregivers of PwD in earlier stages were less likely to endorse comfort care as their current preference (no impairment & MCI: OR = 0.17, p<0.001; mild: OR = 0.36, p<0.001), and caregivers of patients with no or mild impairment had 5.24 times the odds of indicating the need for further discussions of treatment preferences (p = 0.02) than caregivers of severe stage patients. Lastly, if caregivers already had ACP‐related conversations with PwD, they were 2.67 times more likely to prefer comfort care for end‐stage disease (p = 0.007).

**Conclusion:**

Our analyses show the importance of providing early dementia‐specific education for PwD and their caregivers on end‐of‐life care. Our findings also highlight ACP as a dynamic and continuous process throughout the disease course.

